# 

**Published:** 1888-01-14

**Authors:** 


					PRICE ONE PENNY. /
NO. 68; Vol. Ill-
January 14th, 1888.
[Registered at the General Post Office
as a Newspaper.]
Per Annum, 6s. 6d., post free.
Monthly Parts, fld.; post free, 7id.
Ditto par Ana,, 7s. 6d. post frea.
rd
gsap
TilHi
A Weekly Institutional Journal of
^sdsJjC^anJIfirrrick
Science, flfteMdne, anb lpbUantbcop^.

				

